# Paradoxical venous air embolism detected with point-of-care ultrasound: a case report

**DOI:** 10.1186/s13089-022-00265-7

**Published:** 2022-05-18

**Authors:** Hector Andres Ruiz Avila, Hans Fred García-Araque, Estivalis Acosta-Gutiérrez

**Affiliations:** 1 Cuidado Crítico, Hospital Universitario Nacional de Colombia, Bogotá D.C, Colombia; 2 Departamento de Medicina Interna, Facultad de Medicina, Universidad Nacional de Colombia, Bogotá, Colombia; 3 Universidad Militar Nueva Granada, Bogotá D.C, Colombia; 4 Hospital Militar Central, Bogotá D.C, Colombia; 5 Cuidado Crítico, Hospital Universitario Nacional de Colombia, Bogotá D.C, Colombia; 6 Instituto de Investigaciones Clínicas, Universidad Nacional de Colombia, Bogotá, Colombia

**Keywords:** Case report, Air embolism, Point-of-care ultrasound, Stroke

## Abstract

Venous air embolism (VAE) is an uncommon event consistent in the entrainment of air from any communication between the environment and the venous vasculature that could occur during central venous catheter (CVC) manipulation, and might trigger circulatory shock within minutes depending on the amount of air embolized. We present a case of a critical care patient who presented sudden clinical hemodynamic deterioration after the removal of central venous catheter. Hemodynamic evaluation with point-of-care ultrasound (POCUS) showed bubbles in both right and left heart cavities wherewith air embolism facilitated by heart septal defect was suspected. Therefore, the patient was reintubated, supported with vasopressors and a new CVC was inserted to proceed with air aspiration. Shortly after, the patient’s hemodynamic status improved in terms of vital signs stabilization. 6 h after the event with optimal perfusion markers and diminished sedation, the patient showed left hemiparesis therefore a cerebral magnetic resonance (MRI) was also performed showing hyperintensity in the right precentral gyrus, so ischemic stroke without hemorrhagic transformation diagnosis was made, because of paradoxical embolism. This case report demonstrates the value of POCUS application as a diagnostic tool in the hemodynamically unstable patient.

**Supplementary Information:**

The online version contains supplementary material available at 10.1186/s13089-022-00265-7.

## Background

Venous air embolism is the entrainment of air from any communication between the environment and the venous vasculature [1]. It is an uncommon entity but it may cause systemic effects and even significant morbidity and mortality depending on the amount of volume embolized [2, 3]. This complication occurs mostly during or after invasive and surgical procedures such as otorhinolaryngological and neurosurgical procedures, but also during the administration of pressurized infusions or central venous catheter manipulation [2–4].

To generate venous air emboli, there must be a venous pressure gradient that favors air migration through a catheter; this situation is typically established in circumstances in which the central venous pressure (CVP) is lower than the atmospheric pressure like in deep inspiration, hypovolemia and assumption of a semi-upright or sitting posture [5, 6]. When air enters the venous system, it can migrate along different pathways: the usual way is through the right chambers and pulmonary circulation; in case of existing a right-to-left shunt, air could travel to arterial circulation (paradoxical embolism); and finally and less frequently air could ascend retrogradely to cerebral venous circulation [5].

Some references argue that more than 5 mL/kg or air embolized could immediately cause an air-lock scenario yielding to right heart failure usually followed by cardiac arrest. Other lower volumes may be related to a variety of signs and symptoms ranging from dyspnea, wheezing, and coughing to chest pain in awake patients. In those patients who are anesthetized, these symptoms would be subtle and therefore the physician must be aware of changes like a steep drop in the end tidal carbon dioxide (etCO_2_), arterial oxygen saturation (SO_2_), blood pressure (BP), heart rate (HR), or variations in ST segment and T wave in ECG [1, 7].

## Case report

A 54-years old male was admitted to the ICU due to severe ARDS secondary to SARS COV-2 infection. He was receiving a protective invasive mechanical ventilation strategy as well as medical treatment for COVID-19 according to the national guidelines from the Colombian Association of Intensive Care including a CVC insertion for drugs and fluids administration. After the 6th day, he was weaned successfully and extubated without any complications and consequently the CVC was removed. Shortly after, the patient suffered clinical deterioration with hypotension (90/40 mmHg), decrease of the oxygen saturation up to 80% with tachycardia and tachypnea requiring respiratory support with a face mask connected to the mechanical ventilator and vasopressors. Considering these sudden changes in patients hemodynamic status and taking into account COVID-19, increased thromboembolic risk [8], besides SARS CoV2 highly contagious nature which may limit patients access to diagnostic imaging due to hemodynamic instability or transfer difficulties [9]; an emergency focused transthoracic ultrasound echocardiography (FATE) was done, as one of multiple protocols of point-of-care ultrasound (POCUS), starting at the subcostal fourth chamber view and followed by the parasternal long-axis view [10]. The ultrasonographic remarkable findings were an impairment of the global systolic function with dilatation of the right ventricle and the presence of gas bubbles moving between right and left heart chambers (Additional file 1: Video S1, Additional file 2: Video S2, Additional file 3: Video S3, Additional file 4: Video S4, Additional file 5: Video S5, Additional file 6: Video S6). Based on clinical evaluation and with this ultrasonographic evidence, the suspected etiology of his shock status was a venous gas embolism with global ventricular systolic dysfunction. Paradoxical embolism was considered based on the presence of bubbles in left chambers during FATE evaluation. This suggested the high probability of the existence of an intracardiac shunt with the risk of a concomitant acute cerebrovascular event.

The initial therapeutic approach was inotropic and vasopressor therapy (milrinone, noradrenaline), invasive ventilatory support, and air bubble aspiration with a new venous central catheter (right internal jugular) with patient turned into a left lateral decubitus position. Shortly after, the patient's hemodynamic status improved in terms of vital signs stabilization and the decrease of vasopressors doses. A second FATE protocol was performed 20 min later showing a better right and left ventricular function with no bubbles into the heart and no other big ultrasonographic findings (Additional file 7: Video S7).

**Fig. 1:**
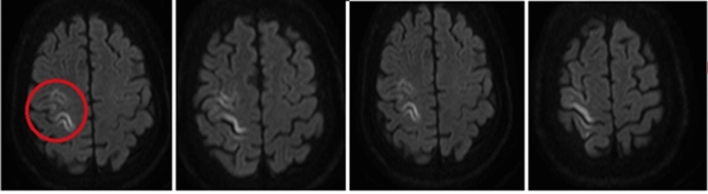
Ischemic stroke: nuclear magnetic resonance image (MRI) showed hyperintensity in this location evaluated in FLAIR: red circle

Six hours after the event with optimal perfusion markers and diminished sedation, the patient showed left hemiparesis studied with a cerebral CT scan that was found without abnormalities. However, with the persistence of mild limitation in upper limb mobility the next day a cerebral magnetic resonance (MRI) was also performed showing hyperintensity in the right precentral gyrus, so ischemic stroke without hemorrhagic transformation diagnosis was made (Fig. 1).

A comprehensive echocardiogram was performed 2 days later by a cardiologist who did not find any atrial or ventricular septal defect. However, with the evidence of bubbles in the right heart in the FATE, he performed an additional transesophageal echocardiogram (TEE) with the maneuver of the bubble contrast being positive. Echo found a thinned interarticular septum with a patent foramen ovale of 0.6 cm which explains the acute neurological symptoms. The Hemodynamics Cardiology Department performed a percutaneous closure of septal defect, according to the high risk of a new event for paradoxical embolism. Clinical evolution was successful with no neurological sequelae remaining and the patient was discharged a few days later.

## Conclusions

Venous air embolism in removal procedure of CVC has an incidence about 0.2–1%, when this complication is suspected, a diagnostic test to visualize the air bubbles should be carried out immediately. For this purpose, ultrasonography is very sensitive at identifying air bubbles that may be undetectable on CT scan [2, 4, 5]. Transthoracic and transesophageal echocardiogram may detect a vascular air volume as small as 0.05 mL/kg and 0.02 mL/kg, respectively [11]. A great advantage of POCUS applications is that ultrasound is a readily available bedside tool that could be used quickly for non-quantitative variables, allowing the identification of signs that may suggest cardiopulmonary compromise secondary to gas emboli migration such as dilatation of the right ventricle and inferior vena cava’s diameter. In severe cases, it would also detect abnormalities in the ventricular wall motion or right ventricular dysfunction [5, 6, 11, 12]. Any time that bubbles are found in both cardiac chambers, the clinician must consider the possibility of left side embolism with the risk of a cerebrovascular acute event, but also the mesenteric or renal arterial tree compromise. In this case, the patient developed a clinical and imagenological stroke with complete recovery and no sequelae. Under the clinical suspicion of cerebral air embolism, diagnosis must be confirmed with images such as the CT SCAN, which could be normal in the next hours of the event like in this case and thus completing cerebral studies with a MRI may be needed [5].

Once VAE diagnosis is made, definitive therapy can be considered, especially if the patient is hemodynamically unstable or complicated by end-organ damage [5]. In case of hemodynamic instability, some studies have suggested that removing the air emboli from the right ventricle outflow tract, and hence maintaining circulation, can be made by increasing central venous pressures with intravenous fluids and patient positioning in Durant’s position (head low and right up) [1]. However, the ideal position assumed by an affected patient remains controversial as recent animal studies have demonstrated a lack of improvement despite relocating air bubbles to the apex of the right ventricle by means of postural changes [5]. Another strategy might be intermittent jugular venous compression, in order to prevent further air entry [1].

In most patients, therapy includes support measures like oxygen supply and in case of life-threatening respiratory failure, mechanical ventilation, vasopressors, and volume resuscitation. Hemodynamically unstable patients and those with end-organ damage or neurological deficits should be treated with definitive therapy such as hyperbaric oxygen, which may be delivered after a significant delay and still provides benefit [2, 3]. However, if this therapy is unavailable, aspiration of air directly from the circulation using intracardiac catheter aspiration with a multiple side-hole catheter such as a 5-French pigtail catheter or even direct aspiration with the tip of a previously positioned CVC approximately 1–3 cm above the sinoatrial node, may be a useful and safe treatment [1–3].

This case report demonstrates the value of POCUS application as a diagnostic tool in the hemodynamically unstable patient done by the attending physician beside the patient. Point-of-care ultrasound is time sensitive to get all the information relevant to help the clinician in the diagnosis and decision-making process, helping to rule out other differential diagnoses and performing the specific treatment; all aiming to decrease morbidity and mortality in these critical scenarios. Specifically, FATE protocol can be used as a screening and monitoring tool for significant cardiopulmonary pathologies specially in admitted patients with acute respiratory symptoms, with the potential of ruling in life-threatening conditions; and evaluate basic hemodynamic determinants such as preload, afterload, contractility, compliance, and relaxation [13, 14].

Particularly during coronavirus pandemic point-of-care ultrasound have been proved useful in early identification of shock mechanisms, assess changes in cardiac, renal or pulmonary function over time through quick routine examinations, perhaps in lieu of heart and lung auscultation, which can be challenging in critical care settings because of pronation, ambient noise, and personal protective equipment or could imply needing of patient mobilization in case of quantitative diagnostics approaches [15].

This tool, widely introduced in the last 4 decades in different settings like in the ICU, OR or the ER, has been well developed by many scientific societies with fantastic efforts to regularize and standardize the training and the practice of ultrasound applications.

We have the commitment to continue using and teaching this mandatory tool for all the clinicians who will be taking care of critical patients in critical scenarios.

Focused sonography of the heart, lungs, and deep veins is fast, highly feasible, and able to establish pathologic conditions in many admitted patients with acute respiratory symptoms. The clinical potential of focused sonography in an ED is as a tool for ruling in life-threatening conditions; at least as important, because of the high NPV, is its value as a standard rule-out examination in patients with respiratory symptoms.

## Supplementary Information

**Additional file 1: Video S1.** Bubbles in right atrio and ventricle, with paradox septum movement in subxiphoid view.

**Additional file 2: Video S2.** Ventricular contractility in short parasternal view.

**Additional file 3: Video S3.** Bubbles in right atrio in subxiphoid view.

**Additional file 4: Video S4.** Bubbles in right atrio in subxiphoid view.

**Additional file 5: Video S5.** Right ventricular collapse and bubbles in left ventricle in subxiphoid view, suggesting septal defect.

**Additional file 6: Video S6.** Right ventricular collapse in subxiphoid view.

**Additional file 7: Video S7.** Four chamber view, showing improvement in biventricular function and no evidence of bubbles into the heart.

## Data Availability

All data generated and analyzed during this study are included in this published article.

## Abbreviations

CVC: Central vein catheter
CVP: Central vein pressure
ICU: Intensive Care Unit
MRI: Nuclear magnetic resonance imaging
POCUS: Point-of-care ultrasound
TTE: Transthoracic echocardiogram
VAE: Venous air embolism
